# Oncolytic tanapoxvirus expressing FliC causes regression of human colorectal cancer xenografts in nude mice

**DOI:** 10.1186/s13046-015-0131-z

**Published:** 2015-02-19

**Authors:** Steven J Conrad, Mohamed El-Aswad, Esaw Kurban, David Jeng, Brian C Tripp, Charles Nutting, Robert Eversole, Charles Mackenzie, Karim Essani

**Affiliations:** Laboratory of Virology, Department of Biological Sciences, Western Michigan University, Kalamazoo, MI 49008 USA; Department of Pathobiology and Diagnostic Investigation, Michigan State University, East Lansing, Michigan USA

## Abstract

Colorectal cancers are significant causes of morbidity and mortality and existing therapies often perform poorly for individuals afflicted with advanced disease. Oncolytic virotherapy is an emerging therapeutic modality with great promise for addressing this medical need. Herein we describe the *in vivo* testing of recombinant variants of the tanapoxvirus (TPV). Recombinant viruses were made ablated for either the *66R* gene (encoding a thymidine kinase), the *2L* gene (encoding a TNF-binding protein), or both. Some of the recombinants were armed to express mouse chemotactic protein 1 (mCCL2/mMCP-1), mouse granulocyte-monocyte colony stimulating factor (mGM-CSF), or bacterial flagellin (FliC). Tumors were induced in athymic nude mice by implantation of HCT 116 cells and subsequently treated by a single intratumoral injection of one of the recombinant TPVs. Histological examination showed a common neoplastic cell type and a range of immune cell infiltration, necrosis, and tumor cell organization. Significant regression was seen in tumors treated with virus TPV/Δ*2L*/Δ*66R*/*fliC*, and to a lesser extent the recombinants TPV/Δ*2L* and TPV/Δ*66R*. Our results suggest that oncolytic recombinants of the TPV armed with activators of the innate immune response may be effective virotherapeutic agents for colorectal cancers in humans and should be explored further to fully realize their potential.

## Introduction

The projected toll exacted by colorectal cancer in humans (hCRC) in 2014 is more than 50,000 deaths, with nearly 100,000 new diagnoses in the United States alone [1,2]. Globally, it is the second-most common cancer in women (after breast cancer) and the third-most common in men (after lung and prostate cancers, respectively) [3]. More than 50% of those stricken by hCRC will experience metastatic sequelae to the liver or, less often, to the lungs [4,5]. Established therapies are often inadequate for patients with advanced disease (stages III or IV, lymph node positive and/or metastatic) where five-year survival rates fall to less than 20% [6]. In the United States the five-year survival rate for patients with all stages of hCRC improved by 14% between 1977 and 2006, from 51% to 65% [6]. This improvement is an aggregate effect of better detection techniques which resulted in earlier detection, improvements in therapeutic efficacy, and a slightly reduced incidence [2]. Still, the grim statistics cited earlier hold true, and new treatments and treatment modalities are desperately needed.

The idea that replication-competent viruses could be used to kill tumors *in vivo* was formally stated over a century ago (reviewed in [7]), but the first modern (*i.e*., genetically engineered) oncolytic virus (OV) was reported relatively recently [8]. Progress in the design of OVs has been rapid [9], and replication-competent oncolytic variants of several viral families have been tested against hCRC and other cancerous growths in clinical trials. These include the adenoviruses [10,11], herpesviruses [12,13], poxviruses [14,15] and reoviruses [16].

Each family of viruses has a unique biology and replication strategy, and this uniqueness presents a distinct set of advantages and disadvantages from the standpoint of OV design. The poxviruses have several inherent qualities that make them well-suited for use as OVs, and have been described as “oncolytic battleships” [17]. This is because poxvirus genomes are able to accommodate a large amount of added genetic material [18], and because all poxviruses come with a built-in array of offensive and defensive capabilities (reviewed in [19]). Poxvirus genomes encode a variety of immunomodulatory proteins devoted to hiding infection-associated cell-surface epitopes from host immunesurveillance, the inhibition and evasion of some host immune and inflammatory responses, and the disruption of signals from the extracellular environment by means of virally-encoded peptides which mimic or neutralize host cytokines and cytokine receptors [19-24].

The OVs described in this study are recombinant variants of the tanapoxvirus (TPV), a member of the genus *Yatapoxvirus*. Several features of the TPV make it an attractive candidate for oncolytic virotherapy. Infected humans experience only a mild and self-limiting febrile illness, in part because TPV infection is normally confined to peripheral areas of the body [25]. Apart from areas in equatorial Africa (where it is endemic) humans are immunologically naïve to TPV [26,27]. Additionally, TPV has never been observed to transmit from person to person, a highly desirable safety feature in an oncolytic virus.

Ablation of the viral thymidine kinase (TK) is a common strategy to increase oncoselectivity in several OV types, especially the herpesviruses and poxviruses [8,28]). This is because TK activity in neoplastic cells is constitutively high, due to the action of cellular TK1. High TK activity in neoplastic cells is in contrast to normal cells, where TK activity levels peak during the S phase of the cell cycle and are nearly undetectable at other times [29-31]. Cellular TK activities are significant from an OV design perspective because the cellular TK1 catalyses a critical step in nucleotide synthesis, the conversion of thymidine to thymidine monophosphate [29]. For this reason, cancerous cells express TK1 throughout the cell cycle, and as a result tend to have large cytoplasmic pools of thymidine monophosphate available at all times. This differential activity has been exploited to provide a degree of cancer cell selectivity in *tk*-ablated OVs. Successful examples of this strategy include the vaccinia virus (VACV) recombinants GLV-1 h68 [32,33].

OVs which express transgenes intended to increase their oncolethality are referred to as “armed” [34,35]. Some of the TPV recombinants described herein were armed to express either mouse (m) granulocyte-monocyte colony stimulating factor (mGM-CSF), mouse macrophage chemotactic protein 1 (mCCL2, also referred to as mMCP-1), or bacterial flagellin (FliC, the product of the *fliC* gene in *Salmonella enterica*). Although mammalian immune system is interdependent and integrated, both cytokines (GM-CSF and CCL2) and bacterial flagellin (FliC) primarily stimulate the innate immune system. Hence, there appears to be no distinct advantage in testing the therapeutic transgenes, described in this study, in syngeneic or transgenic models at this stage. Nude mice have an intact and functional innate immune system, but lack a functional adaptive immune response. Though the adaptive immune response in nude mice is ineffective, immature CD3+ T cells have been reported [36]. We, therefore, concluded that the use of nude mice in this study is valid to report the impact of innate immunity on oncolytic virotherapy.

CCL2 is a pleiotropic chemokine and is known to exert effects beyond its primary effect, including bone remodeling and bone disease [37] and the atopic response [38,39]. CCL2 has been reported to enhance host anti-tumor immune response elicited by some OVs [40].

Expression of GM-CSF by an OV, or its introduction to the tumor environment by other means is a well-established strategy in OVs, anticancer vaccines and vaccine adjuvants [41-43]. There are many examples of the use of GM-CSF as part of a strategy to engineer GM-CSF-expressing tumor cells (autologous or allogeneic) and re-introduce them into the tumor environment [44,45]. Many replication-competent OVs have been armed to express GM-CSF [46-49].

Polymerized flagellin is the main component of the bacterial flagellum [50,51]. The flagellin used in this study was the product of the *Salmonella enterica* serovar *typhimurium* gene, *fliC*. FliC and other bacterial flagellins are cognate ligands of the toll-like receptor 5 (TLR5) [52,53], and are strong activators of the innate immune response in mammalian cells via MyD88-dependent intracellular signaling and, ultimately, the activation of transcription factor NFκB [54]. TLR 5 activation has been shown to substantially increase necrosis and lead to tumor regression [55]. Here we describe the oncolytic ability of TPV recombinants to regress hCRC xenografted tumors in athymic nude mice.

## Methods

### Cells, reagents and viruses

OMK, HCT 116, COLO 205, SW1463 and WiDr cells were purchased from the American Type Culture Collection (ATCC product numbers CRL-1556, CCL-247, CCL-222, CCL-234 and CCL-218, respectively). OMK (Owl Monkey kidney) cells were used for all virus amplification and viral titrations. All cell lines were propagated in complete growth medium consisting of Dulbecco’s Modified Eagle’s Medium (Gibco/Life Technologies) supplemented with 10% (vol/vol) fetal bovine serum (FBS) (Atlanta Biologicals), 2 mM L-glutamine (Sigma-Aldrich) and 50 μg/ml gentamicin sulfate (AMRESCO). After virus infection all cell monolayers were maintained in maintenance medium which was identical to growth medium except that the concentration of FBS was reduced to 2%. All cells were incubated at 37°C in a 5% CO_2_ atmosphere. Cell counting and cell viability assays were done with an Improved Neubauer hemacytometer using 0.2 % (wt/vol) trypan blue in a normal saline solution. Wild-type TPV (Kenya strain) was originally a gift from Dr. Joseph Esposito (Centers for Disease Control, Atlanta, GA, USA). It was genetically modified in the laboratory of G. McFadden to express the fluorescent reporter enhanced green fluorescent protein (EGFP), but with no other modifications.

### The p2KO vector

For the construction of the p2KO vector we used the commercially-available plasmid cloning vector pBluescript II KS(+) as starting material. This cloning vector is a high-copy number plasmid which features ampicillin selection and has a multiple cloning sites which contains many useful and unique restriction endonucleases sites. We inserted two identical synthetic early/late promoters [56] between the unique Xba I and Xma I sites and the Xma I and EcoRI sites, in each case using long primers to include the 52-bp promoter and the small sequence between the 3′- end of the promoter and the start codon on the transgene (expressed transgene or fluorescent reporter). Flanking sequences from the wild-type TPV genome were inserted between unique Sac I and Not I restriction sites (left flank) and unique EcoR I and Hind III restriction sites (right flank). Flanking regions were ligated into the p2KO vector between the Sac I and Not I restriction sites on the 5′- (left) flank, and between the EcoR I and Hind III restriction sites on the 3′- (right) flank. In this way the p2KO expression cassette could be guided to a specific point in the viral genome by the use of viral genomic flanking sequences, resulting in a targeted ablation of the desired gene(s) with the simultaneous expression of a fluorescent reporter and (if desired) an expressed transgene (in this study m*CCL2*, m*GM*-*CSF* or *fliC*). The relevant primers are documented in Table 1.

**Table 1:** **Primers used in the construction of the p2KO ablation**/**insertion vector**

**Primer name**	**Sequence**
left flank	
66R L SacI (f)	5′-AATGGATCACATAAAG*GAGCTC*TTAACG-3′
66R L NotI (r)	5′- CAGAAAACAT*GCGGCCGC*ATATAATCT-3′
right flank	
66R R EcoRI	5′-GGAGATGAACAAGAAATA*GAATTC*ATAGG-3′
66R R HindIII	5′- CTGTTCTTTATCAC*AAGCTT*CTATCGGGTG-3′
m*GM*-*CSF*	
hmGMCSF BamHI (f)	5′-TAGGCCTG*GGATCC*GATCCACCGGTCGCCACCATGTGGCTGCAGA-3′
mGMCSF XmaI (r)	5′-CTCATCAATGTATCTTATCAT*CCCGGG*CTAGCT-3′
m*CCL2*/*MCP*-*1*	
mMCP-1 BamHI (f)	5′-TAGGCCTG*GGATCC*GATCCACCGGTCGCCACCATGCAGGTCCCTG-3′
mMCP-1 XmaI (r)	5′-CGGCGATC*CCCGGG*AGATACTAGTTCAC-3′
*fliC* (*S. typhimurium*)	
FliC BamHI (f)	5′-ACCCGG*GGATCC*TCTAGAAATAATTTTG-3′
FliC XmaI (r)	5′-GGAGCTCGAA*CCCGGG*TCCTTAAC-3′
M13 (f)	5′-TGTAAAACGACGGCCAGT-3′
M13 (r)	5′-CAGGAAACAGCTATGACC-3′
*66R* ablation verification	
66R int (f)	5′- CGGTATCAAATTGCTAGGTATACTTGC-3′
66R int (r)	5′- CTCCAATTCGTTTAGAAAACGATGCG-3′
*2L* ablation verification	
2L int (f)	5′- CCATTGCATCCTTCAGAACAAG-3′
2L int (r)	5′- GCATAACTTTAAAATATAATTATACTGTTACG-3′
PCR template control	
136R int (f)	5′- GTATTTATGTACTGTTTCAACTAACAAAAGC-3′
136R int (r)	5′- CCTTTAGGTGTTAGGATATATCAATTATACAG-3′

Where applicable the inserted restriction endonucleases sites are indicated by italicized text and start codons within the forward primer and/or the stop codons within the reverse primer are shown by gray shading. The 66R int (f/r) and 2L int (f/r) primer sets are verification primers used to verify 66R and/or 2L ablations, while the 136R gene is present in all viruses and is used as a positive control for the quality of the template DNA in PCR reactions.

The m*CCL2* cDNA clone ORF was purchased as an ORF-bearing plasmid (Sino Biological, Incorporated). The cDNA clone ORF of m*GM*-*CSF* was a gift from Dr. Grant McFadden. The m*CCL2*, m*GM*-*CSF* and *fliC* ORFs were amplified from their vectors by PCR and given BamHI and XmaI restriction sequences on the 5′- and 3′- termini of the product amplicons. They were ligated into the p2KO poxvirus vector in the expressed transgene insertion site. All resulting plasmids were confirmed by DNA sequencing.

**Figure 1:**
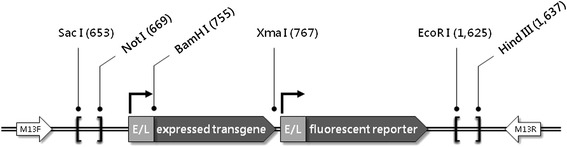
**The poxvirus ablation** **/insertion vector p2KO.** Separate but identical poxvirus early/late synthetic promoters allowed for simple and targeted ablation of selected gene(s) with the simultaneous expression of the fluorescent reporter mCherry with or without the expression of an additional expressed transgene. The left and right flanks are bounded by pairs of unique restriction sites (Sac I and Not I for the left flank, EcoR I and Hind III for the right flank). The expressed transgene ORF is bounded by unique a 5′- BamH I and a 3′- Xma I restriction site. These allow for the simple and directional ligation of PCR amplicons bounded by the appropriate restriction sites. Numbers in small type refer to nucleotide positions within the p2KO vector.

**Figure 2:**
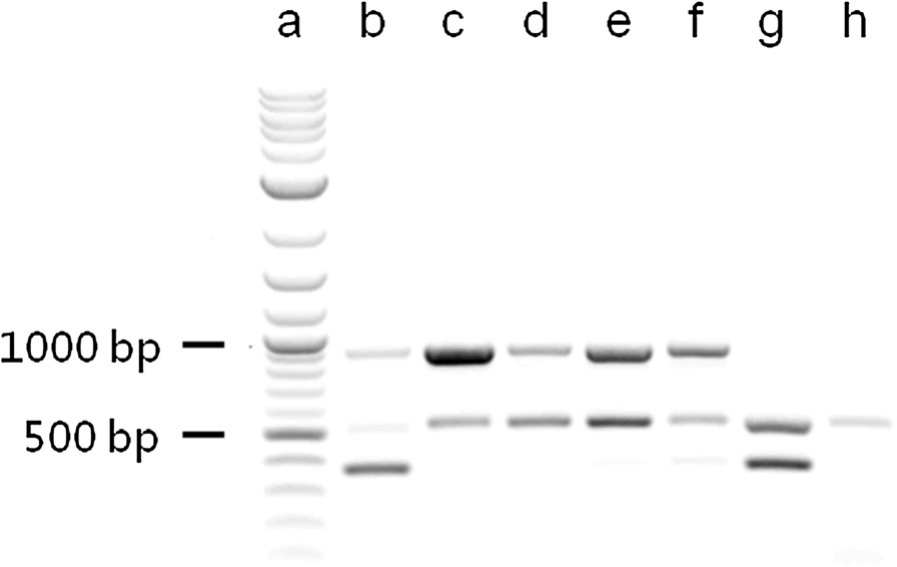
**Verification of ablation in recombinant TPVs.** Agarose gel electrophoresis (1%) were done to verify the ablation of the viral *2L* and *66R* genes. Each viral DNA was probed for sequences internal to the knocked-out *2L* and *66R* region. An ablated gene will show no band. The *2L* region predicted amplicon is 904 bp; the *66R* region predicted region is 379 bp; a region within the 136R gene was used as a control and had a predicted amplicon length of 531 bp. Lane a: molecular weight marker 2-log ladder (New England Biological); Lane b: TPV/*egfp*; Lane c: TPV/Δ*66R*/*mGM*-*CSF* (*66R* is ablated); Lane d: TPV/Δ*66R*/*mCCL2* (*66R* is ablated); Lane e: TPV/Δ*66R*/*fliC* (*66R* is ablated); Lane f: TPV/Δ*66R*/*fliC* (*66R* is ablated); Lane g: TPV/Δ*2L* (*2L* is ablated); Lane h: TPV/Δ*2L*/Δ*66R*/*fliC* (both *2L* and *66R* are ablated).

The p2KO vector was intended to provide a rapid and reliable way to simultaneously ablate any desired TPV gene(s) and replace the ablated gene(s) with an expressed transgene (if desired) and an expressed fluorescent reporter. It was completely modular in that the ORFs in either the expressed transgene or the fluorescent reporter insertion sites could be easily removed and replaced with any other ORFs (Figure 1). The overall sequence of the base vector (*i.e*., with the mCherry fluorescent reporter but without an inserted expressed transgene) was verified by DNA sequencing of an amplicon produced by PCR amplification of the region between the M13 forward and reverse primer binding sequences. The insertion of ORFs encoding m*CCL2*, m*GM*-*CSF* or *fliC* was verified by DNA sequencing of the p2KO plasmid vector to ensure correct placement and orientation before they were used in the transfection/infection procedure. The recombinant viruses were verified to be deleted for the core regions of *2L*, *66R*, or both by agarose gel analysis of PCR products using recombinant viral DNA as template (Table 2). Primer sets (Table 1) which amplified sequences internal to the ablated regions in the *66R* and *2L* genes were used to verify the absence of these genes in the recombinant TPVs made. A similar primer set which amplified a region of the *136R* gene was used to verify the suitability of the DNA preparation for PCR amplification. The predicted amplicon size for the *66R* internal primer set is 379 bp; the predicted amplicon size for the *2L* primer set is 904 bp; and for the *136*R control primer set the amplicon size is 531 bp. A recombinant virus with an ablated gene failed to produce a PCR product when probed for that particular gene with these primer sets (Figure 2).

**Table 2:** **Viruses made using the p2KO ablation**/**insertion vector**

**TPV recombinant**	**Gene ablated**	**Gene added**	**Reporter** **(s)**
TPV/*egfp*	---	EGFP	
TPV/Δ*66R*	*66R*	---	mCherry
TPV/Δ*66R*/m*GM*-*CSF*	*66R*	m*GM*-*CSF*	mCherry
TPV/Δ*66R*/m*MCP*-*1*	*66R*	m*MCP*-*1*	mCherry
TPV/Δ*66R*/*fliC*	*66R*	*fliC*	mCherry
TPV/Δ*2L*	*2L*	---	mCherry
TPV/2L/Δ*66R*/*fliC*	*66R*, *2L*	*fliC*	EGFP, mCherry

All viruses expressed the fluorescent reporter mCherry with the exception of TPV/*egfp*, which was modified to express fluorescent reporter EGFP but had no other genetic modifications. “Gene added” refers to an ORF added and expressed other than the fluorescent reporter. The TPV/Δ*2L*/Δ*66R*/*fliC* double-ablated recombinant TPV expressed both EGFP and mCherry.

The p2KO expression cassette (left and right flanks, plus the intervening ORFs and the promoters) was transferred to the viral genome through a homologous recombination double-crossover event during transfection/infection. During transfection/infection, poxvirus genomes present in the cytoplasmic space of infected cells were in close proximity to the transfected p2KO vector, whose flanking sequences allowed for a targeted double-crossover homologous recombination event. During the double-crossover event, the region between the flanking sequences on the p2KO vector was transferred to the viral genome, simultaneously ablating the intervening viral sequence and resulting in a recombinant viral genome which contains the fluorescent reporter and (if desired) an additional ORF, both of which are now driven by synthetic early/late promoters derived from VACV.

### Transfection/infection

The transfection/infection procedure used to produce the recombinant viruses in this study has been described previously [57-59]. Briefly, OMK cells were transfected using jetPRIME transfection reagent (PolyPlus Transfection SA) at a concentration of 1 μl transfection reagent per μg of purified p2KO plasmid vector according to the manufacturer’s protocol. At approximately five hours post transfection, OMK cell monolayers were inoculated with 1 plaque-forming unit (pfu) per cell of wild-type TPV-Kenya strain (no fluorescent reporter expressed). At five days post-inoculation the infected monolayers were scraped with a cell scraper on ice and the lysates were processed by three cycles of freezing and thawing at −80°C followed by 15 seconds of sonication at 4°C. Samples were serially diluted and plated onto freshly-seeded OMK cell monolayers at approximately 90% confluence and overlaid with maintenance medium containing 0.5% methylcellulose. Fluorescent, well-separated plaques were picked and each pick subjected to at least three rounds of plaque purification to produce a virus preparation which contained no visible wild type (non-fluorescent) plaques. Samples were considered pure if no wild-type plaques were visible in culture and no relevant fragments of wild-type TPV genomic DNA were detectable by PCR.

Because it was necessary to ablate two discrete genetic loci (the *2L* and *66R* genes) the TPV/Δ*2L*/Δ*66R*/*fliC* virus was made using an additional iteration of the transfection/infection procedure. Using a plasmid (generously provided by J. Barrett) which contained a single VACV early/late synthetic promoter driving expression of EGFP, we inserted sequences flanking the *66R* gene, resulting in a *66R*-ablation vector. This vector was used in the transfection/infection procedure with wild-type TPV (Kenya) to make TPV/Δ*66R*/*egfp*. Selection was enabled by the expression of EGFP against a non-fluorescent background. After 3X plaque purification, this EGFP-expressing virus was used in place of the wild-type TPV in a second transfection/infection with a p2KO ablation/insertion vector which contained sequences flanking the *2L* gene, the *fliC* ORF, and the mCherry fluorescent reporter. Selection was enabled by the expression of mCherry-expressing plaques against a background of EGFP expression.

### Confirmation of viral transgene expression

Verification of FliC expression was done by Western blot of virus infected cell lysates. Proteins were transferred to a PVDF membrane (Millipore) and probed with an anti-FliC monoclonal antibody (BioLegend) at a 1:2000 dilution (vol/vol). Powdered milk 5% (wt/vol) was used as the blocking agent. The secondary antibody was a monoclonal anti-mouse IgG conjugated to horseradish peroxidase (Abcam), used at a 1:2500 dilution. Visualization was by enhanced chemiluminescence (ECL) (Thermo Scientific/Pierce). TPV recombinants containing the p2KO vector’s fluorescent reporter but without any other expressed transgenes (empty vectors) and uninfected cells served as controls in these experiments. Verification of mCCL2 and mGM-CSF expression was done by Luminex multianalyte cytokine detection assay (University of Maryland cytokine core laboratory), both in cell lysates and supernatants from virus infected cells. Samples for analysis were prepared by infecting semi-confluent OMK cell monolayers in 60 mm tissue culture dishes with TPV/Δ*66R*/m*CCL2*, TPV/Δ*66R*/m*GM*-*CSF* or TPV/Δ*2L*/Δ*66R*/*fliC* using 10 pfu/cell. Supernatant (3 ml/dish) and cytoplasmic extracts were prepared at the indicated times post-infection. Uninfected cell lysates and cells infected with virus not expressing mCCL2, mGM-CSF, or FliC served as controls for confirmation of expression experiments.

### Cell density determinations

Four human colorectal cancer cell lines and the OMK cell control were separately inoculated into 12-well plates (3 wells per cell line) such that one day later the cells were 90% confluent. Each well was trypsinized, counted and scored for viability by trypan blue exclusion. This was done to ensure that pfu/cell for each cell line would be accurate.

### Virus titration

To assay the number of viable virions present in a sample, a plaque assay was used as previously described [60]. Briefly, virus samples were subjected to three rounds of freezing and thawing at −80°C, sonicated for 15 seconds on ice, serially diluted in maintenance medium and inoculated onto nearly confluent OMK cell monolayers in 6-well plates. Virus was allowed to adsorb at room temperature with gentle rocking for one hour. The inoculum was then removed and each well washed two times with 1 ml of pre-warmed (37°C) maintenance medium. After washing, 2 ml of overlay medium was added and the infected OMK monolayers incubated for 10 days at 37°C. The overlay medium was then removed and monolayers were stained (0.1% crystal violet in 37% formaldehyde). Plates were washed with distilled water, dried in air, and plaques were counted on a light box. Each experiment was independently repeated three times.

### Animals

Male athymic nude (Nude-*Foxn1*^*nu*/*nu*^) mice (Harlan Laboratories) were received at four weeks of age and allowed to acclimate for one week before experimentation. Mice were individually housed in clear polycarbonate cages under a 12 hour/12 hour light/dark cycle. Food and water was available *ad libitum*. All animal housing conditions, manipulations and treatments were performed in accordance with the protocols approved by the Institutional Animal Care and Use Committee of Western Michigan University (IACUC protocol number 13-07-01).

### Choice of cell line for tumor xenografts in nude mice

Before initiating *in vivo* studies in athymic nude mice, we determined which hCRC cell line had the highest viral productivity when infected with TPV/*egfp*. TPV/*egfp* was assayed for its ability to replicate in four hCRC-derived cell lines: HCT 116, WiDr, SW1463 and COLO 205. OMK cells were used as a positive control. Each cell line was seeded into 12-well tissue culture plates and 0.1 pfu/cell TPV/*egfp* inoculated into each well. Lysates were collected at 4 days post-infection and assayed by plaque assay, as described earlier. Each experiment was independently repeated three times.

### Tumor induction and measurement

Tumors were produced in athymic nude mice by subcutaneous injection of 5 × 10^6^ HCT 116 cells on the dorsal surface, approximately above the first lumbar vertebra. Each injection was followed by an assessment of viability by trypan blue exclusion to ensure that the cells were viable at and after the time of injection. Once visible, tumors were measured using a digital caliper (Pittsburgh, model 6ZBTMCO) along the major axis (length), minor axis (width) and z dimension (height), which were substituted into the formula volume = (length) × (width) × (height) × (π/6). When tumor size reached or surpassed 75 mm^3^ the animal was randomly segregated into the control group or one of the seven experimental virotherapy groups.

### Virotherapy of HCT 116 xenografts in nude mice

Each treatment group was composed of five or six tumor-bearing athymic nude mice. A single virotherapeutic injection was administered once tumor volume reached or exceeded 75 mm^3^. Virotherapeutic injections were given intratumorally as a single injection of 5 × 10^6^ pfu suspended in 100 μl of crude OMK cell lysate diluted in normal saline. The control consisted of animals which received the HCT 116 cells but experienced only a mock virotherapeutic injection (100 μl of vehicle only). This group is referred to as the mock virotherapy group. Mouse weights and tumor volumes were measured and recorded at three-day intervals thereafter. Data were collected for 13 time points over a total of 39 days. To control for unanticipated inflammation or other injection effects produced by the administration of the virotherapeutic injection, a vehicle control group was used.

### Immunohistochemistry preparation

Formalin fixed specimens were processed, embedded in paraffin and sectioned on a rotary microtome at 4 μm. Sections were placed on adhesive slides and dried at 56°C overnight. The slides were subsequently deparaffinized in xylene and hydrated through descending grades of ethanol to distilled water. Slides were placed in Tris buffered saline pH 7.5 for 5 minutes. Slides were then heat treated (in a rice steamer for 30 minutes followed by 10 minutes at room temperature, or in Pascal Pressure Cooker for 15 minutes at 125°C followed by 80°C for 5 minutes and then 30 minutes at room temperature on countertop) or underwent enzyme induced epitope retrieval (10 minutes at 37°C). These processes were followed by subsequent rinses and blocking for endogenous peroxidase using 3% hydrogen peroxide/methanol bath (1:4 ratio) for 20 minutes at room temperature followed by running tap and distilled water rinses. Following pretreatments, standard avidin – biotin complex staining steps were performed at room temperature on a Dako Autostainer rinsing with Tris buffered saline + Tween 20 between all staining steps in protocol. Slides were blocked for non-specific protein binding with Normal Goat or Rabbit Serum (Vector Labs – Burlingame, CA, USA) for 30 minutes. Endogenous Biotin was blocked by incubation in Avidin D (Vector) and D-Biotin (SigmaAldrich – St.Louis, MO, USA) for 15 minutes in each reagent. Primary antibodies, rabbit polyclonal anti-CD3 (AbD Serotec, Raleigh NC), rat anti-mouse – F4/80 (Abcam Cambridge, MA), and rabbit polyclonal anti–caspase 3 (Abcam, Cambridge MA) were diluted in normal antibody diluent (Scytek – Logan, UT, USA) and incubated for 1 hour at room temperature. Biotinylated rabbit anti–rat IgG H + L (mouse absorbed) (Vector) diluted 5 ug/ml or goat anti-rabbit and IgG H + L (Vector) diluted 11 ug/ml for 30 minutes and R.T.U. VectaStain Elite ABC reagent (Vector) for an additional 30 minutes. Reactions were developed with Nova Red (Vector) for 15 minutes; followed by counter-stain in Gill 2 hematoxylin (Richard Allen – Kalamazoo, MI, USA) for 15 seconds, differentiated in 1% aqueous glacial acetic acid and rinsed in running tap water. Slides were then dehydrated through ascending grades of ethanol, cleared through several changes of xylene, and cover-slipped using flotex permanent mounting medium.

### Histological analyses

In order to assess the validity of this cell transplant system the slides prepared above were used to determine the presence of tumor cells, as well as the nature of the immune response. Several key criteria were subjectively assessed; these criteria included the presence and form of the neoplastic cell line, evidence of cell death, extent and form of accumulating and infiltrating host inflammatory cells, degrees of fibrosis, and overall distribution of the infiltrating host cells within the tissues.

### Microscopy and image capture

Slides were reviewed using brightfield microscopy on a Nikon Eclipse 80i microscope (Tokyo, Japan) with Nikon Plan 10x objective lens (N.A. 0.25) and Plan Fluor 40x objective lens (N.A 0.75). Images were taken using MetaMorph (Sunnyvale, CA, USA) imaging software and Qimaging (Surrey, BC, Canada) MicroPublisher Color Camera using the 10x objective lens. Scale bars were applied to the images using MetaMorph using calculated length per pixel specific for the camera and the objective lens. All scale bars were set to 200 μm.

### Statistics

To assess treatment effects each experimental group was compared to the mock virotherapy control using the Mann–Whitney *U* test (sometimes referred to as the Wilcoxon rank-sum test). Virotherapeutic treatment was considered to have produced a significant therapeutic effect if the average tumor volume at a specific time point and within a group was significantly reduced when compared to the mock-virotherapy control. A significance level of p < 0.05 was used throughout the study.

## Results

### The p2KO vector and recombinant viruses

During the transfection/infection, punctuate expression of the fluorescent reporter was evident in the OMK cell monolayer by five days post-transfection, indicating gene expression from the p2KO vector in the cytosolic compartment of cells infected with wild-type TPV. In control cultures which were transfected with the p2KO vector but not subsequently inoculated with wild-type TPV, no fluorescence was observed, demonstrating that expression of viral genes did not occur in the absence of viral infection. After 3X plaque purification the absence of wild-type TPV DNA in the viral sample was verified by PCR using the recombinant viral genomic DNA as the template. All viral DNA samples were probed for the presence of the ampicillin resistance gene, which was not detected in any recombinant virus (data not shown). This indicates that all of the recombinant viruses resulted from a double-crossover event rather than a single-crossover event. The TPV/Δ*2L*/Δ*66R*/*fliC* virus required a second iteration of transfection/infection and plaque purification.

**Figure 3:**
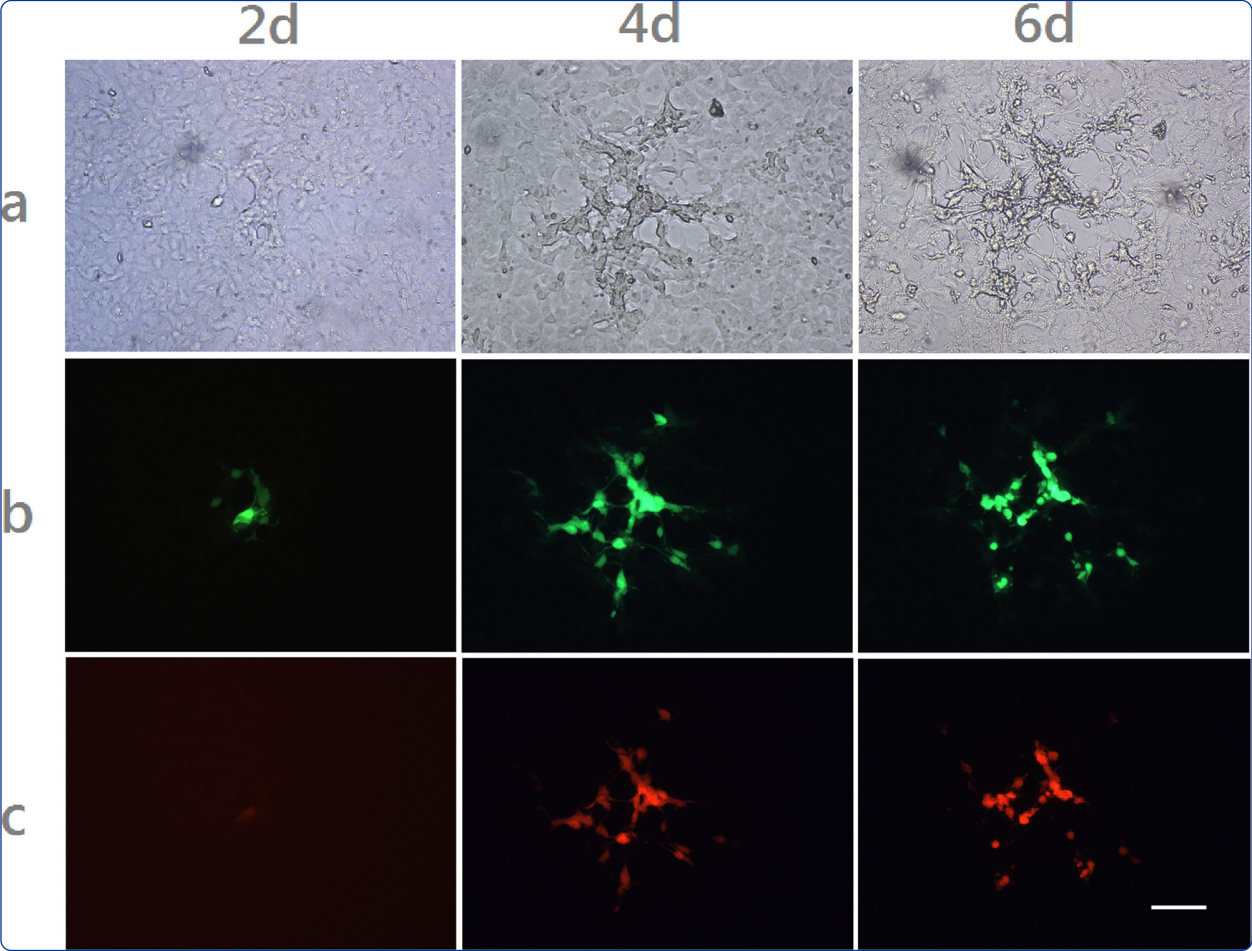
**The recombinant virus TPV**/**Δ** ***66R***/**Δ** ***2L***/***fliC*** **in culture.** The plaque morphology associated with the TPV as visualized using the fluorescent reporters EGFP and mCherry present in the recombinant ablated for both *66R* and *2L*, the TPV/Δ*66R*/Δ*2L*/*fliC*. **(a)** A phase contrast view of the plaque in an OMK cell monolayer at days 2, 4 and 6 post-infection, visualized in white light. **(b)** The same plaque viewed at excitation and emission wavelengths which allow EGFP to be visualized. **(c)** The same plaque viewed at excitation and emission wavelengths which allow mCherry to be visualized. Microscope used was a Nikon Diaphot, total magnification 400X, exposed for 1.5 seconds. Image capture was performed with QImaging color camera using MetaMorph software (version 7.6.0.0, Molecular Devices). Scale bar = 100 μm.

The selection of recombinant viral plaques and the subsequent visualization of viral infection in cultured cells was greatly facilitated by the inclusion of the fluorescent reporters mCherry (λ_ex_/λ_em_ = 587 nm/610 nm) or enhanced green fluorescent protein (λ_ex_/λ_em_ = 475 nm/509 nm). Furthermore, the use of two fluorescent reporters made it possible to identify and isolate the double-ablated recombinant virus with the *fliC* insertion (TPV/Δ*2L*/Δ*66R*/*fliC*). A viral plaque at 2, 4 and 6 days post-infection produced by the TPV/Δ*2L*/Δ*66R*/*fliC* in an OMK cell monolayer is shown in Figure 3, and demonstrates the simultaneous expression of the brilliant orange-red and green color associated with mCherry and EGFP, respectively. A total of seven recombinant viruses were generated. These include: TPV/*egfp*, TPV/*Δ66R*, TPV/*Δ66R*/m*GM*-*CSF*, TPV/*Δ66R*/m*MCP*-*1*, TPV/*Δ66R*/*fliC*, TPV/*Δ2L*, TPV/*Δ2L*/*Δ66R*/*fliC* (Table 2).

### Confirmation of viral transgene expression

**Figure 4:**
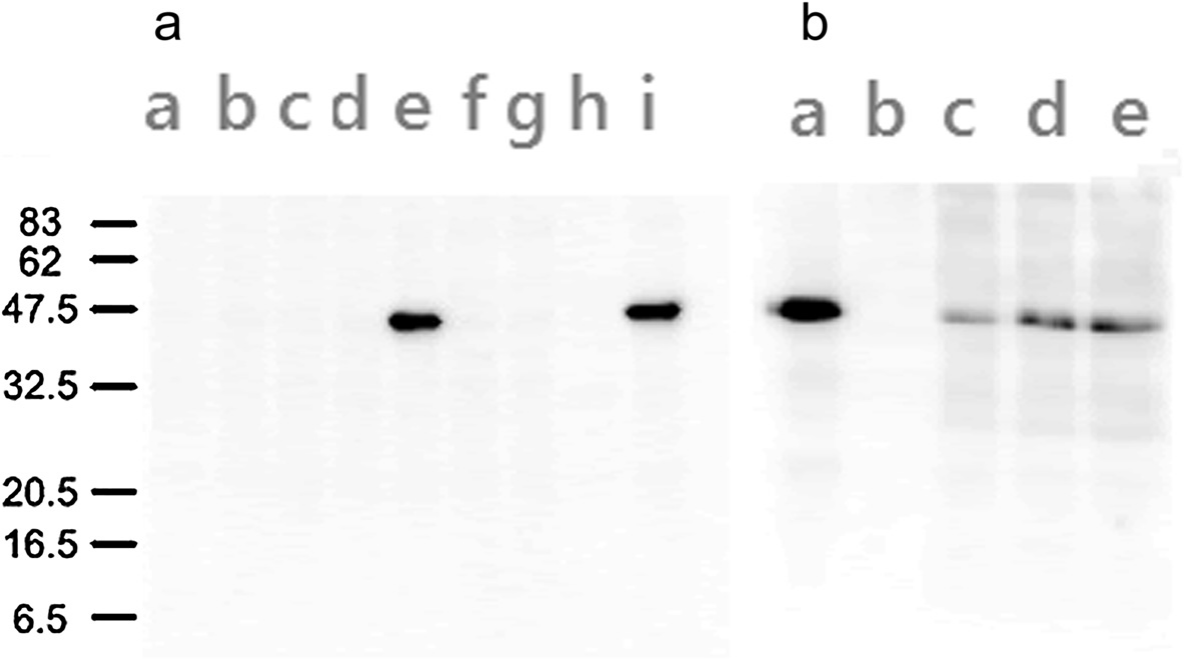
**FliC expression in recombinant TPV** **/Δ** ***66R/*** ***fliC*** **TPV** **/Δ** ***2L*** **/Δ** ***66R/*** ***fliC*** **.** The viruses TPV/Δ*66R*/*fliC* TPV/Δ*66R*/Δ*2L*/*fliC* were inoculated into 60 mm dishes onto semi-confluent OMK cells at 10 pfu/cell and cytoplasmic extracts at 3, 5 and 6 days post-infection were subjected to 12% SDS-PAGE and Western blot analysis using a monoclonal antibody against FliC and a secondary anti-mouse HRP conjugated antibody. Visualization of bands was by ECL. Gel **(a)** lanes **(a)** mock infected, day 0; **(b)** mock infected, day 3; **(c)** wild-type TPV; **(d)** TPV/Δ*66R*, the “empty vector” control; **(e)** TPV/Δ*66R*/*fliC*; **(f)** TPV/egfp; **(g)** TPV/Δ*66R*/m*GM*-*CSF*; **(h)** TPV/Δ2L; **(i)** TPV/2L/Δ*66R*/*fliC*. Gel **(b)** lanes **(a)** FliC (53 kDa) purified from *S. enterica*, the positive control; **(b)** mock infected, day 6; **(c)** TPV/Δ*66R*/Δ*2L*/*fliC*, day 3 post-infection; **(d)** TPV/Δ*66R*/Δ*2L*/*fliC*, day 5 post-infection; **(e)** TPV/Δ*66R*/Δ*2L*/*fliC*, day 6 post-infection.

Before the start of *in vivo* experiments, we first demonstrated that the inserted ORFs were expressed in cells infected with the recombinant viruses TPV/Δ*66R*/m*CCL2*, TPV/Δ*66R*/m*GM*-*CSF* and TPV/Δ*2L*/Δ*66R*/*fliC*. OMK monolayers in 60 mm dishes were inoculated with the relevant recombinant virus (10 pfu/cell) and assayed for transgene expression in cell lysates and culture supernatants as described in the Methods. A Western blot of infected cell lysates from OMK cells inoculated TPV/Δ*2L*/Δ*66R*/*fliC* was probed with a monoclonal anti-FliC antibody (Figure 4). A single band was observed, identical to the FliC positive control (purified FliC), as expected. The intensity of this band gradually increased between day three and day six post-infection. The FliC transgene was not detected in cells infected with the recombinant TPV containing only the empty vector and/or any virus not expressing FliC.

Since the inserted mCCL2 and mGM-CSF ORFs contained the eukaryotic secretion signal sequence, infected cells were assayed for the presence of mCCL2 and mGM-CSF both in infected cell supernatants and cell lysates. Both transgenes were highly expressed and present in large amounts in infected cell supernatants (4.9 ng/ml mCCL2, and greater than 10.0 ng/ml mGM-CSF) as assayed by ELISA. Both mGM-CSF and mCCL2 were only weakly detectable or undetectable in cytoplasmic extracts of infected cells, and in control uninfected cells or in cells infected with viruses not expressing these transgenes. These results indicated that both mCCL2 and mGM-CSF were secreted from infected cells, as anticipated, while FliC accumulated within the cytoplasm of infected cells over the course of the infection.

### Cell density determinations and virus replication

Each cell line used in this study had its confluent density (cells/cm^2^) determined at near confluence (as described in the Methods). Our control cell line, OMK, had a confluent density of approximately 1.0 × 10^5^ cells/cm^2^. The densities determined for the human colorectal cancer cell lines were as follows: HCT 116 had a confluent density of approximately 1.4 × 10^5^ cells/cm^2^; COLO205 had a confluent density of approximately 6.9 × 10^5^ cells/cm^2^; SW1463 had a confluent density of approximately 4.5 × 10^5^ cells/cm^2^; WiDr had a confluent density of approximately 2.5 × 10^5^ cells/cm^2^. These values were used to calculate the number of pfu to use when inoculating these cell lines for virus susceptibility assays. All cell lines supported virus replication. Almost all cells in the monolayers were lysed by day 10 following virus infection, as expected since TPV is a cytolytic virus.

Before initiating *in vivo* studies in athymic nude mice, we determined which hCRC cell line had the highest viral productivity when infected with TPV/*egfp*, as described in the Methods. OMK cells were the best host cells, allowing the production of approximately 3 × 10^6^ progeny pfu/well (9.5 cm^2^). Of the hCRC cell lines tested, HCT 116 produced the most progeny virions with an average yield (n = 3) of approximately 7 × 10^5^ progeny pfu/well. We therefore chose the cell line HCT 116 for the *in vivo* phase of this study.

### Virotherapy HCT 116 xenografts in nude mice

**Figure 5:**
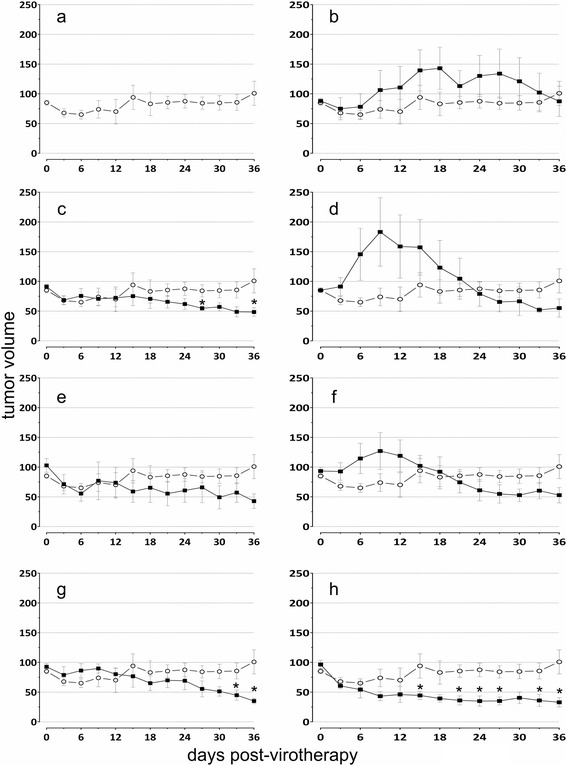
**Virotherapy.** Tumors were induced by subcutaneous injection of 5 × 10^6^ HCT 116 cells onto the dorsal surface of athymic nude mice. In each group n = 5 or 6 mice. Mice were randomly segregated into the control or experimental groups when tumor size reached 75 mm^3^. In each graph the y-axis is tumor volume (mm^3^) and the x-axis is time (days post virotherapeutic injection). **(a** **)** A single mock injection containing 100 μl of vehicle only or recombinant virus was administered at day 0 and tumor volume was measured at three-day intervals. Tumor volume was calculated using the formula (length) × (width) × (height) × (π/6). Average tumor volume is shown (black filled squares for each experimental group). Bars show the standard error of the mean (±1 SEM). Points indicated with an asterisk (*) are significantly reduced from the control (p ≤ 0.05). **(a)** Mock virotherapeutic injection (vehicle only, open gray circles). This is the group to which all experimental groups were compared to test for OV virotherapeutic effect upon average tumor volume. **(b)** TPV/*egfp*; **(c)** TPV/Δ*66R*/m*MCP*-*1*; **(d)** TPV/Δ*66R*/m*GM*-*CSF*; **(e)** TPV/Δ*66R*/*fliC*; **(f)** TPV/Δ*66R*; **(g)** TPV/Δ*2L*; **(h)** TPV/Δ*2L*/Δ*66R*/*fliC*.

In order to evaluate the oncolytic potential of TPV recombinants, we induced HCT 116 tumors in athymic nude mice (Nude-*Foxn1*^*nu*/*nu*^). Viability counts of HCT 116 cells from rapidly-dividing cultures demonstrated that > 99% were viable at the time of injection. Tumors generally reached 75 mm^3^ within one to three weeks after implantation of HCT 116 cells. Comprehensive virotherapeutic results are shown in Figure 5. Virotherapy with TPV/Δ*66R* (Figure 5f), TPV/Δ*2L* (Figure 5g), and TPV/Δ*2L*/Δ*66R*/*fliC* (Figure 5h) all produced significant reductions in tumor size at two or more time points when compared to the mock-injected controls. TPV/Δ*2L*-treated tumors were significantly smaller than mock-injected tumors at two time points, 33 days (a 47.6% reduction) and 36 days (a 65.2% reduction) post-treatment. TPV/Δ*66R*-treated tumors were significantly smaller than mock-injected tumors at two time points, 27 days (a 34.9% reduction) and 36 days (a 52% reduction) post-treatment. TPV/Δ*2L*/Δ*66R*/*fliC*-treated tumors showed a robust and durable virotherapeutic effect and were significantly reduced in volume when compared to mock-injected tumors at six time points, 15 days (a 56.1% reduction), 21 days (a 62.0% reduction), 24 days (a 63.8% reduction), 27 days (a 59.5% reduction), 33 days (a 55.3% reduction) and 36 days (a 69.6% reduction) after virotherapeutic treatment.

### Histological validation of the tumor model

**Figure 6:**
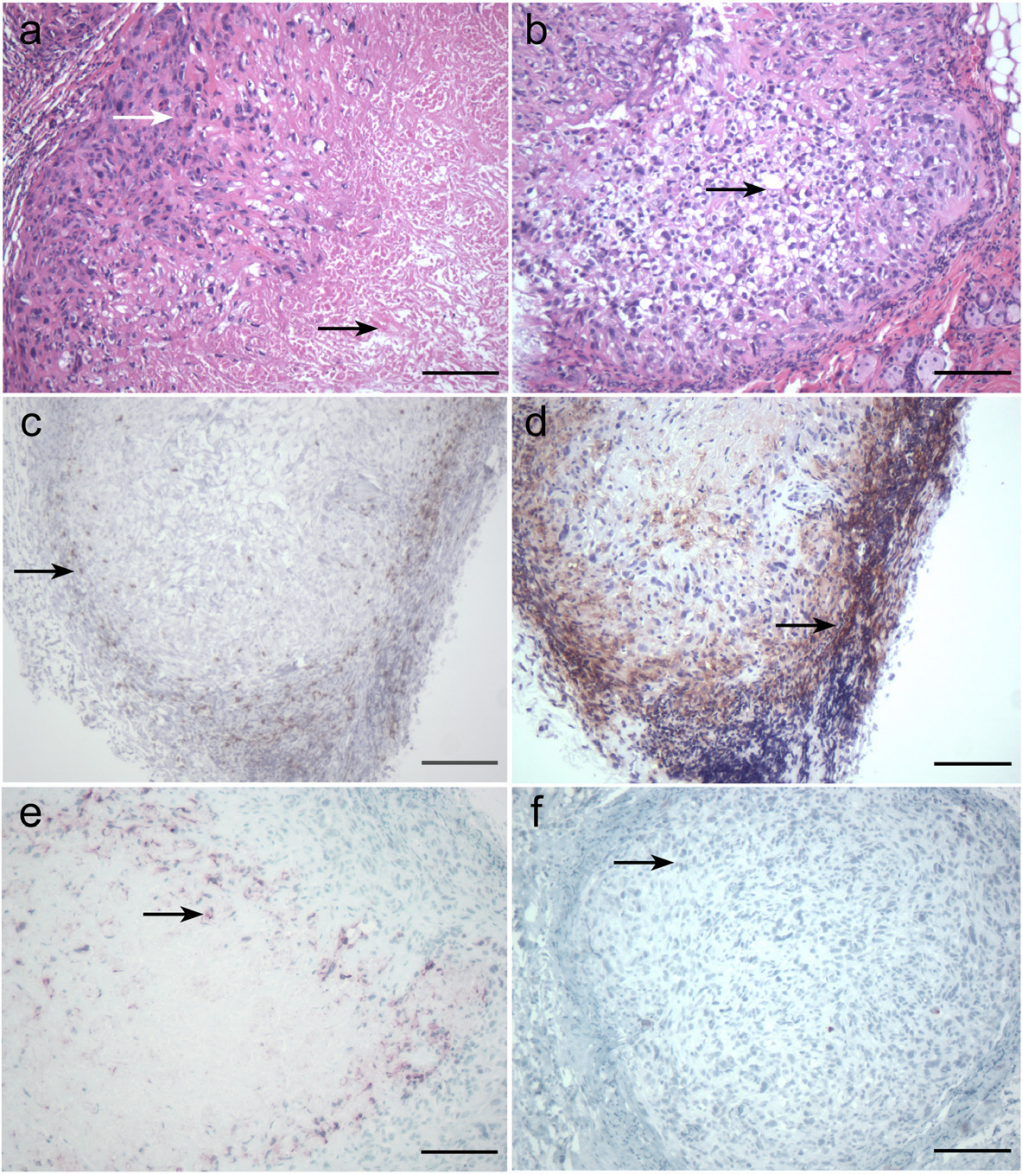
**Histology of tumors. (a)** H&E stained tumor with healthy neoplastic cell ring (white arrow) around the necrotic tumor center (black arrow); **(b)** H&E stained tumor with areas of tumor cell vacuolation and althered morphology (black arrow); **(c)** CD3 stained tumor showing lymphocytic infiltration in host-tumor interface (black arrow); **(d)** F4/80 stained tumor showing macrophage response (black arrow) including infiltration into the tumor mass; **(e)** Caspase stained tumor showing areas of caspase activity (black arrow) within tumor associted with the pale-staining necrotic area; **(f)** Caspase staining of a tumor free of cellular necrosis tumor showing the lack of caspase activity associated with healthy neoplastic cells (black arrow). All bars 200 μm.

The primary neoplastic cell type was seen to be present in all samples examined; the cellular and nuclear morphology of this cell type varied distinctively between samples. The degree of necrosis and evidence of damage to this cell type varied from group to group and was clearly associated with caspase positivity and a range of morphological forms including nuclear pyknosis, cell ghosting, swelling, and vacularisation of the cells, were evident (Figure 6). The size of the tumors, as well as the proportion of viable to necrotic tumor cells also varied between samples. There were varied degrees of lymphocytic (CD3) and macrophage (F480) host responses to the presence of the tumor masses, with the greatest accumulation of these two cell types at the interface of the host and tumor tissue areas. Macrophages and scattered lymphocytes were seen invading the main tumor tissue areas in a relatively small number of samples.

## Discussion

Two of the major problems associated with the use of viruses for the treatment of cancers include (a) the absence of immune tolerance for an oncolytic virus, which will ultimately result in a strong antiviral immune response, and (b) person-to-person transmission of the therapeutic OV. In the absence of an effective immune tolerance strategy, it will be necessary to have a number of oncolytic viruses available for a serial treatment strategy and TPV should, therefore, be considered as a potential addition. The safety issue mainly deals with human-to-human transmission. Again, unlike some other potential oncolytic viruses, TPV has not been reported to transmit from person to person.

Wild-type viruses from some families possess a degree of native oncoselectivity, most notably the reoviruses, vesicular stomatitis Indiana virus, and the Newcastle disease virus [61]. This selectivity arises from their ability to exploit defects in the cellular antiviral defenses commonly found in cancerous cells but absent in non-transformed cells. Examples of such defects include inappropriately activated *Ras* and the resulting reduction and/or loss of PKR activity exploited by reovirus [62-64] and the reduction and/or loss of the type I interferon response and subsequent failure to activate the PKR antiviral response [65-68], which is responsible for the cancer cell selectivity shown by the vesicular stomatitis Indiana virus.

In viruses without significant native oncospecificity, genetic engineering has been employed to increase cancer cell selectivity. Although some preference for cancerous and/or transformed cells has been shown in oncolytic variants of VACV [69], wild-type poxviruses are not generally considered to have a high degree of native oncospecificity. Ablation of the thymidine kinase gene in VACV has been shown to increase its cancer cell selectivity [70,71]. We therefore made TPV recombinants in which the thymidine kinase gene (*66R*) was ablated in an attempt to increase the oncoselectivity of the TPV. Although mice are not normally receptive animal hosts for TPV replication, it was important to demonstrate that the ablation of the *66R* gene did not result in a non-replicative virus in permissive cells (such as HCT 116 tumor cells).

We also made viruses ablated for the *2L* gene, the gene product of which is 2L (formerly referred to as TPV-gp38), a protein with TNF-binding activity [21-23]. In a normal TPV infection, secreted 2L acts to blunt the host inflammatory and antiviral immune response by binding to and effectively reducing the amount of TNF present. While this is a desirable outcome for the virus, when TPV is used as an OV it may be advantageous to increase, rather than decrease, the amount of inflammation at the tumor site. Currently the literature is divided regarding the role of inflammation upon tumor destruction and clearance [72,73]. In a hypothetical human patient whose tumor is treated with an OV based upon TPV, we hypothesize that the ablation of the *2L* gene may result in an effective increase in TNF concentration at the tumor site, which may ultimately act to increase tumor clearance. Because we have previously shown that the TPV 2L binds to human TNF but not mouse TNF [22], the ablation of 2L in some of the recombinant viruses described here was not expected to be a significant factor in tumor clearance. Additionally, we did not find any significant differences in TNF levels in the sera of any mice used in this study when assayed by ELISA (results not shown). We have nevertheless included *2L*-ablated recombinants in this study because the testing of an OV in xenografted athymic nude mice is an important step towards further testing in more relevant primate models of cancer virotherapy.

Direct viral cytolysis (rather than tumor cell destruction by the host immune response) is of critical importance to tumor clearance in some cases [74], but viral cytolysis is only one of many factors impinging upon tumor survival and clearance, and immune cell recruitment can also play a critical role [75,76]. Because TPV is a new entrant into the field of oncolytic poxviruses, we tested wild-type TPV expressing EGFP with no other modifications (TPV/*egfp*) against a panel of human colorectal cancer cell lines to select the cell line which allowed the best viral replication, thereby maximizing the effect of direct viral tumor cell lysis. The hCRC cell lines tested for TPV/*egfp* replication were HCT 116, COLO205, SW1463 and WiDr. Although HCT 116 produced fewer progeny virions than the control cell line OMK, HCT 116 was by far the most productive of the hCRC cell lines tested. Many OVs have been characterized in tumors induced with HCT 116 [32]. The expression of TLR-5 has been demonstrated in HCT 116 cells [77]. For these reasons we selected HCT 116 for further characterization of the oncolytic potential of TPV recombinants *in vivo*.

We induced tumors in athymic nude mice with the HCT 116 cell line. One significant difference between our experience and many published studies [78,79] which used the same cell line was that our HCT 116-induced tumor xenografts did not increase in volume to the expected level during virotherapy. However, we did observe the development of multiple secondary tumors in these mice. The precise reason behind this observation is difficult to evaluate. The shedding, high-motility and invasiveness of HCT 116 cells [80] may have been contributing factors. Furthermore, this has been shown in an HCT 116 orthotopic xenotransplant model [81], in which *ex vivo* HCT 116 tumors were subserosally implanted onto the ceca and ascending colon of 32 Balb/c nude male mice, and ultimately metastasized to distant locations, including invasion to liver, lung, as well as both liver and lung. Figure 5a shows the observed tumor development (from the point at which the tumor mass exceeded 75 mm^3^) in athymic nude mice xenografted with 5 × 10^6^ HCT 116 cells and subsequently given only mock virotherapy. The average tumor volume consistently increased until approximately 15 days post virotherapy, at which point its volume stabilized at between 100–200 mm^3^ in most animals. This is in contrast to some previous studies which have shown that untreated HCT 116 tumors in nude mice gradually increase in volume over the same interval, when using similar numbers of HCT 116 cells in the initial xenograft. For example, it has been reported that HCT 116-induced tumors have a doubling time of approximately 8 days [78]. Also, a recent study which examined the VACV as an OV therapeutic against HCT 116 xenografts in nude mice showed HCT 116 tumor growth up to 4000 mm^3^ in a time interval almost identical to ours [32].

Histological analyses of the tumors and associated tissue from all animals showed that the primary neoplastic cell type of the HCT 116 cell line was present in all the samples (Figure 6a). The cellular and nuclear morphology of this cell type varied distinctly between samples (Figure 6a, 6b). The degree of necrosis, or evidence of damage to this cell type, varied from group to group. Some cell death was clearly associated with caspase positivity (Figure 6e, 6f) and took a range of morphological forms, including; nuclear pyknosis, cell ghosting, swelling, and vacularisation (Figure 6b) of the cells. The size of the tumors, as well as the proportion of viable to necrotic tumor cells also varied between samples. There were substantial and varied lymphocytic (Figure 6c) and macrophage (Figure 6d) host responses to the presence of tumor masses, with scattered lymphocytes invading the main tumor tissue in a relatively small number of samples.

The addition of the *fliC* transgene in a double-knockout background (Δ*66R* and Δ*2L*) resulted in the most efficacious OV in this study, producing more significantly-reduced tumor volumes and doing so at earlier times than any other recombinant TPVs tested. As stated above, the single-knockout virus (*66R*/thymidine kinase deleted) which expressed FliC approached but did not achieve a significant level of efficacy. TPV which was Δ*2L* with no other modifications (Figure 5g) or which expressed FliC in a Δ*66R* background (Figure 5e) both trended towards significance but did not approach the efficacy of the TPV/Δ*2L*/Δ*66R*/*fliC* virus (Figure 5g). It may be possible that the absence of 2L somehow enabled the virus to persist longer within the tumor environment, since we detected virus in excised tumors only in the *2L* knockout viruses (data not shown).

If the presence of FliC did indeed contribute to the observed reduction in tumor size, then the mechanism by which this occurred is an open question. As a highly-conserved pathogen-associated molecular pattern (PAMP), bacterial flagellins are important targets for detector molecules involved in immunosurveillance. For example, detection of flagellin by the Nod-like receptor NCLR4 (also known as Ipaf) triggers activation of the Ipaf inflammasome [82] which in turn activates caspase-1 and maturation of the cytokine interleukin 1β (IL-1β) in macrophages [83]. Although the expected amount of FliC produced during the course of an OV infection would be small, even minute amounts of bacterial flagellin (≤5 μg/animal) administered to mice by tail vein injection causes global (i.e., in both organs and plasma) elevations of the cytokines TNF, IL-1β, IL-6, and the chemokine MIP-2 (IL-8), as well as changes in the MEK intracellular signaling pathway [84]. Further, TPV is a cytolytic virus, therefore infected cells are lysed resulting in the release of the virally encoded FliC into the extracellular environment whereby it may trigger the activation of TLR5 and subsequent release of the previously mentioned inflammatory cytokines. Despite the current imperfect understanding flagellin’s mechanism of action in mammalian cells, further research with OVs expressing this potent activator of the innate immune response should be performed. However, we have provided evidence that the activation of the innate immune response by TPV expressing FliC does contribute to the reduction of tumor mass. This observation is significant because the innate immune response is functionally intact in nude mice.

Our results demonstrate that the virus ablated for both *2L* and *66R* which expressed the *fliC* transgene produced a robust and durable therapeutic effect upon HCT 116 tumor xenografts in athymic nude mice. Both of the single-knockout viruses (TPV/Δ*66R* or TPV/Δ*2L*) showed statistically significant reductions in tumor volume at least two time points, and in each case the observed significant reduction in tumor volume was temporally distant from the point of virotherapeutic inoculation. Both viruses definitely appeared to trend towards an effect at these later points. Indeed, with the exception of the TPV/*egfp* virus, all recombinant viruses tested appeared to produce some degree of tumor ablation, but only the viruses mentioned above produced a statistically significant effect. It may be that large intra-group variability masked our ability to detect virotherapeutic potency in this experiment. If this is indeed the case, then perhaps all of the recombinant viruses tested here are largely reliant upon direct cytolysis to promote tumor regression in this model, but the expression of FliC may expedite the response. More animals per group and a more typical rate of tumor growth should alleviate this problem in future studies. Since the T cell-dependent adaptive immune response is severely impaired in nude mice, the experiments described in this study suggests that the innate immune response has potentially contributed to the reduction of tumor burden. Additionally, Rhee et al. [55] have clearly demonstrated that injecting soluble FliC into human colorectal tumors xenografted in nude mice resulted in significant reduction in tumor mass. This effect was not observed when MyD88 and/or TLR-5 knockout cells were used. Furthermore, vaccinia virus GLV-1 h153 another poxvirus, has recently been shown to effectively reduce the human colorectal tumor burden in nude mice orthotopically xenografted [85]. Taken together our results strongly support the notion that TPV armed with FliC has potential to be an effective oncolytic virus which mediates its antiviral efficacy via both viral cytolysis and innate immune activation through TLR-5.
